# Standardized positive controls for detection of norovirus by reverse transcription PCR

**DOI:** 10.1186/1743-422X-8-260

**Published:** 2011-05-26

**Authors:** Sung-Geun Lee, Soe-Hyun Lee, Seung-Won Park, Chang-Il Suh, Weon-Hwa Jheong, SeHwan Oh, Soon-Young Paik

**Affiliations:** 1 Department of Microbiology, College of Medicine, The Catholic University of Korea, Seoul 137-701, Republic of Korea; 2 Department of Agricultural Biology, National Academy of Agricultural Science, Rural Development Association, Suwon, 441-707, Republic of Korea; 3 Environmental Infrastructure Research Department, National Institute of Environmental Research, Incheon 404-170, Republic of Korea

## Abstract

**Background:**

Norovirus is one of the most common causes of nonbacterial gastroenteritis in humans. Rapid spread by contaminated food and person-to-person transmission through the fecal-oral route are characteristics of norovirus epidemiology and result in high morbidity in vulnerable patient populations. Therefore, detection of norovirus is a major public health concern. Currently, the most common method for detecting and differentiating among norovirus strains in clinical and environmental samples is reverse transcription PCR (RT-PCR). Standardized positive controls used in RT-PCR assays to detect norovirus are designed to overcome the problem of false-negative results due to PCR inhibitors and suboptimal reaction conditions.

**Results:**

In the current study, four types of RNA transcripts were produced from plasmids: norovirus GI-5 and GII-4 capsid regions with human rotavirus (VP7 gene derived) fragment insertions, and norovirus GI-6 and GII-4 capsid regions with hepatitis A virus (VP1/P2A gene derived) fragment insertions. These size-distinguishable products were used as positive controls under the RT-PCR assay conditions used to detect NoV in stool and groundwater samples. Their reliability and reproducibility was confirmed by multiple sets of experiments.

**Conclusions:**

These standardized products may contribute to the reliable and accurate diagnosis by RT-PCR of norovirus outbreaks, when conducted by laboratories located in different regions.

## Background

Gastroenteritis, also known as "stomach flu", is a major public health concern and causes over 1.8 million deaths worldwide every year in children younger than five. Several studies and diagnostic analyses have shown that noroviruses (NoVs) are the leading cause of acute nonbacterial gastroenteritis. Although the gastroenteritis caused by NoVs is mild and of short duration, it affects persons of all ages and sometimes leads to death. Although severe illness is rare, complications related to dehydration can occur in very young and elderly individuals, and those with weakened immune systems. Depending on whether the dehydration is treated promptly, patients in these groups may face life-threatening risks. Therefore, the detection and monitoring of NoV is an important task for national public health agencies [1,2].

Norovirus is a member of the *Caliciviridae *family and has been classified into five genogroups (GI to GV) based on capsid gene sequence homology. The positive-sense, single-stranded RNA genome sized 7.6 kb contains three open reading frames (ORFs) [3,4]. The NoVs that are the most common causative agents of human infection are in the GI and GII genogroups; each of these genogroups is further divided into 8 and 17 genotypes, respectively [4-6].

When sensitive diagnostic methods first became available, the pathogenic importance of norovirus was not yet known. Since 1972, when NoV was first discovered by immuno-electron microscopic examination of stool samples, several methods for the detection of NoV have been developed [7,8]. Once the sequence of NoV was available, RT-PCR methods of detection were developed. Because current RT-PCR methods are sensitive and specific enough to detect low levels of a broad spectrum of viruses from samples, RT-PCR is an invaluable tool for evaluating outbreaks. The selected target for RT-PCR detection of viruses is usually the RNA polymerase gene, which is conserved among the NoVs. Of the currently available assays for viral detection (electron microscopy, immuno-electron microscopy, serology, and RT-PCR), RT-PCR assay of stool samples is the most widely used clinical detection method.

Although RT-PCR is potentially a fast and reliable method of detecting and typing NoV, it has some drawbacks, such as the possibility of false-negative or -positive results [9]. In the present study, to address the issue of false-negative results and to confirm NoV detection, standard positive-control products that were different in size from the RT-PCR product of the NoV target region were designed. These positive controls were then used in RT-PCR under the same conditions as actual clinical and environmental samples.

## Methods

### Viral RNA preparation and RT-PCR reaction

Stool samples from patients with viral diarrhea (collected and provided by Chungnam University Hospital in 2006 and Changwon Fatima Hospital in 2005) from the Waterborne Virus Bank were used for NoV viral RNA preparation. Samples from Keimyung University Dongsan Hospital and from Chungbuk National University Hospital in 2009 were used for the preparation of viral RNA from human rotavirus (HRV) and hepatitis A virus (HAV), respectively.

One gram of each stool sample was suspended in 9 ml of Dulbecco's phosphate buffered saline, and 140 μl of the suspended sample applied to the QIAmp Viral RNA Mini kit (QIAGEN), in accordance with the manufacturer's instructions.

The viral RNA was eluted with 60 μl of diethyl pyrocarbonate-treated water and then used as the template for RT-PCR. RT-PCR was conducted with the One-Step RT-PCR PreMix kit (iNtRON Biotechnology, Korea) according to the manufacturer's instructions. Briefly, 5 μl of each viral template RNA was mixed with 1 μl (20 pmol) of each of the One-step RT-PCR primers (Table 1), 13 μl of water, and One-step RT-PCR PreMix. The reverse transcription reaction took place at 45°C for 30 min, followed by RNA denaturation at 94°C for 5 min and then 30 cycles of denaturation (94°C for 30 s), annealing (NoV, 55°C for 60 s; HRV, 60°C for 60 s; and HAV, 65°C for 60 s), extension (72°C for 1 min), and final extension (72°C for 5 min), with a Px2 thermal cycler (Thermo Fisher Scientific, USA).

**Table 1:** Primer sets used to clone NoV capsid regions, HRV VP7, and HAV VP1/P2A.

Virus, region	Primers and Sequences (5'→3')	Location	Application
NoV GI Capsid	GI-FIM; CTGCCCGAATTYGTAAATGATGAT	5342-5365^a^	Onestep RT-PCR
	GI-RIM; CCAACCCARCCATTRTACATYTG	5649-5671^a^	Onestep RT-PCR and Semi-nested PCR
	GI-F2; ATGATGATGGCGTCTAAGGACGC	5358-5380^a^	Seminested PCR
NoV GII Capsid	GII-FIM; GGGAGGGCGATCGCAATCT	5049-5067^b^	Onestep RT-PCR
	GII-RIM; CCRCCIGCATRICCRTTRTACAT	5367-5389^b^	Onestep RT-PCR and Semi-nested PCR
	GII-F3M; TTGTGAATGAAGATGGCGTCGART	5079-5102^b^	Semi-nested PCR
HRV VP7	ddrv-1; GGCGCCGCTCYTTTTRATGTATGGTATTGAATTACCAC	6-38^c^	Onestep RT-PCR
	ddrv-2; GGCGCCCTTTAAAATANAYDGADCCWRTYGGCCA	346-373^c^	Onestep RT-PCR
HAV VP1/P2A	HAV-1F; GGCGCCATTCAGATTAGACTGCCTTGGTA	2789-2811^d^	Onestep RT-PCR
	HAV-1R; GGCGCCAGTAAAAACTCCAGCATCCATTTC	3365-3388^d^	
	HAV-2F; CTATTCAGATTGCAAATTACAAT	2886-2908^d^	Semi-nested PCR
	HAV-2R; AACTTCATTATTTCATGCTCCT	3258-3279^d^	

^a^GenBank accession number M87661

^b^GenBank accession number X86557

^c^GenBank accession number HQ392461

^d^GenBank accession number AF268396

The RT-PCR products were analyzed by electophoresis on a 2% agarose gel and then cloned into the pGEM-T Easy Vector (Promega, USA). Plasmid transformation of *E. coli *DH5α was then confirmed by sequencing (Cosmo Genetech, Korea).

### Viral gene manipulation and plasmid construction

Plasmid DNA was prepared from transformed *E. coli *DH5 α with a plasmid DNA mini kit (Real biotech, Taiwan). pGEM-T Easy Vector plasmids containing the NoV fragment (which has a single *Nar*I restriction site in the middle of capsid region) were digested with the restriction enzyme *Nar*I (Roche Applied Science, Germany) and then treated with alkaline phosphatase (Roche Applied Science, Germany). Plasmids containing HRV (VP7 gene derived) or HAV (VP1/P2A gene derived) gene fragments that were cloned with primer sets designed to add *Nar*I recognition sites were also incubated with *Nar*I. The digested HRV and HAV fragments were then purified with a Gel/PCR DNA Mini kit (Real Biotech, Taiwan) from 2% agarose gels. The purified fragments were ligated into the *Nar*I-digested NoV plasmids using a DNA ligation kit (TaKaRa, Japan) and transformed into *E. coli *DH5α. Successful transformation was then confirmed by sequencing (Cosmo Genetech, Korea).

### In vitro RNA transcription

To produce RNA transcripts under the T7 promoter, plasmids were linearized with the restriction enzyme *Spe*I (Roche applied Science, Germany) and transcribed with the MAX large-scale RNA production system with T7 polymerase (Promega, USA). RNA transcripts were then purified with the RNA Cleanup kit (QIAGEN, Germany).

### Application to clinical and environmental samples

Stool samples and environmental sample obtained from the Waterborne Virus Bank were used for NoV viral RNA preparation. Viral RNA was extracted using QIAmp Viral RNA Mini kit (QIAGEN, Germany), in accordance with the manufacturer's instructions. To confirm size-distinguishable products, RT-PCR was conducted with a One-Step RT PCR PreMix kit (iNtRON Biotechnology, Korea). RT-PCR and semi-nested PCR amplication were applied with primer sets (Table 1). We used 5 μl of viral RNA or RNA transcripts as the template and 15 μl of the premixed kit solution. The PCR was carried out in a PCR System Px2 thermal cycler (Thermo Hybaid, Middlesex, United Kingdom) according to the following protocol: one initial RT step at 45°C for 30 min, followed by PCR activation at 94°C for 5 min; followed by 30 cycles of amplification at 30 s at 94°C, 60 s at 55°C and 65°C, and 1 min at 72°C; with a final extension step of 10 min at 72°C. In the case of environmental sample, 2 μl of products from this reaction was used for the templates in the semi-nested PCR with 18 μl of PCR PreMix (iNtRON Biotechnology, Korea) and the primer sets (NV-GIF2/NV-GIRIM for NoV GI; NV GIIF3M/NV-GIIR1M for NoV GII; HAV-2F/HAV-2R for HAV). The nested PCR protocol was as follows: 94°C for 5 min; followed by 25 cycles of amplification at 30 s at 94°C, 30 s at 55°C and 65°C, and 90 s at 72°C; with a final extension step of 10 min at 72°C. The PCR products were run on 1.5% agarose gels, stained with ethidium bromide, and visualized under UV light. The products were extracted from the agarose gel with a QiaQuick PCR purification kit (QIAGEN, Germany), and the sequences were analyzed by Cosmogenetech (Seoul, South Korea).

## Results

### Cloning of RT-PCR amplified NoV, HRV, and HAV genes

**Figure 1:**
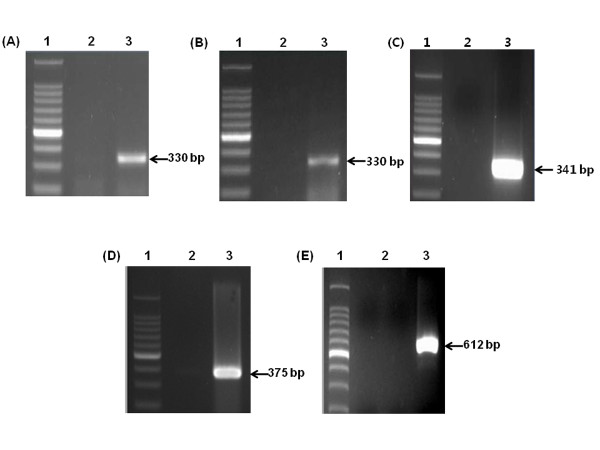
**Transcripts of targeted viral genes produced by RT-PCR prior to insertion into plasmids**. Capsid regions of NoV GI-5 (A), NoV GI-6 (B), NoV GII-4 (C), HRV VP7 (D), and HAV VP1/P2A (E). Lane 1, 100 bp ladder; lane 2, negative controls; lane 3, positive results.

Viral RNA extracted from the stool samples of patient hospitalized from viral diarrhea was used as the template for RT-PCR. The amplified RT-PCR products were then analyzed by 2% agarose gel electrophoresis. The NoV capsid regions GI and GII were 330 bp and 341 bp, respectively. Sequencing analysis of the cloned RT-PCR products indicated that the NoV genotypes were GI-5, GI-6, and GII-4 (Figure 1A, B, and 1C). These three NoV capsid genotypes were cloned into the pGEM-T Easy Vector, and the resulting plasmids were used as backbones for the construction of standardized positive control plasmids.

To create size-distinguishable products, the VP7 and VP1/P2A regions of HRV and HAV, respectively, were amplified with primer sets designed to contain *Nar*I recognition sites and then inserted into pGEM-T Easy Vectors for further manipulation. The RT-PCR products of HRV and HAV from the stool samples had the expected size of 375 bp and 612 bp, respectively (Figure 1D and 1E). The virus from which the HRV fragments originated was classified as the G4[P6] genotype by sequencing.

### Confrimation of standardized positive-control plasmids containing cloned viral gene

**Figure 2:**
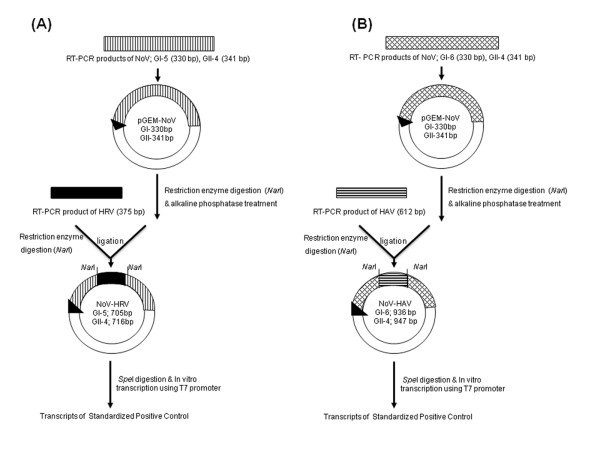
**Strategy to produce standardized positive-control plasmids containing cloned viral genes**. The four types of resulting plasmids were used to produce RNA transcripts. (A) NoV GI-5 + HRV and NoV GII-4 + HRV, (B) NoV GI-6 + HAV and NoV GII-4 + HAV.

As shown in Figure 2, cloned NoV capsid gene-containing plasmids were digested with *Nar*I and then treated with alkaline phosphatase prior to ligation with the HAV and HRV gene fragment RT-PCR products. The four resulting plasmid types contained 705, 716, 936, or 947 bp NoV-plus-insert fragments, as confirmed by sequencing.

### Confirmation of size-distinguishable standardized positive-control

To determine whether they were suitable as positive controls, the RNA transcripts were used as templates in RT-PCR under the same conditions used for environmental and clinical samples. When RT-PCR to detect NoV was performed, the positive control reactions yielded transcripts of the expected size.

**Figure 3:**
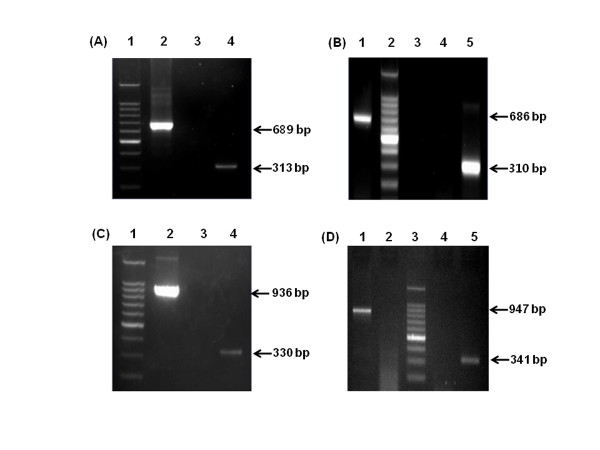
**Confirmation of size-distinguishable standardized positive controls under the same RT-PCR conditions as clinical and environmental samples**. (A) NoV GI-5 + HRV and environmental sample: lane 1, 100 bp ladder; lane 2, standardized positive control; lane 3, negative control; lane 4, environmental sample. (B) NoV GII-4 + HRV and environmental sample: lane 1, standardized positive control; lane 2, 100 bp ladder; lane 3, negative control; lane 4, negative control; lane 5, environmental sample. (C) NoV GI-6 + HAV and clinical sample: lane 1, 100 bp ladder; lane 2, standardized positive control; lane 3, negative control; lane 4, clinical sample. (D) NoV GII-4 + HAV with clinical sample: lane 1, standardized positive control; lane 2, negative control; lane 3, 100 bp ladder; lane 4, negative control; lane 5, clinical sample.

The First, For the detection of NoV in environmental sample, RT-PCR amplication was carried out with size-distinguishable standardized positive-control, NoV positive stool samples (Genogroup I and II), and D.W as negative control. Two NoV Positive sample were detected as shown by the 313 bp and the 310 bp amplified band (Figure 3(A), lane 4 and Figure 3(B), lane 1). In addition, it was observed that the 689 bp and the 686 bp size-distinguishable standardized positive-control amplified band was detected (Figure 3(A), lane 2 and Figure 3(B), lane 1). Negative control was not detected.

The Second, for the detection of NoV in stool sample, was carried out with size-distinguishable standardized positive-control, NoV positive stool samples (Genogroup I and II), and D.W as negative control. Two NoV Positive sample were detected as shown by the 330 bp and the 341 bp amplified band (Figure 3(C), lane 4 and Figure 3(D), lane 5). In addition, it was observed that the 936 bp and the 947 bp size-distinguishable standardized positive-control amplified band was detected (Figure 3(C), lane 2 and Figure 3(D), lane 1). Negative control was not detected.

### Detection of HRV and HAV in clinical and environmental samples

**Figure 4:**
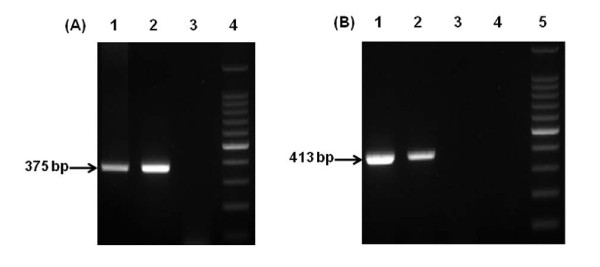
**Detection of HRV and HAV using standardized positive controls under the same RT-PCR conditions as clinical and environmental samples**. (A) Detection of HRV in clinical sample: lane 1, HAV standardized positive; lane 2, clinical sample; lane 3, negative control; lane 4, 100 bp ladder. (B) Detection of HAV in environmental sample: lane 1, HAV standardized positive; lane 2, environmental sample; lane 3, negative control; lane 4, negative control; lane 5, 100 bp ladder.

For the detection of HRV in clinical sample, RT-PCR amplification was carried out with standardized positive-control, HRV positive stool sample, and D.W as negative control. All of both standardized positive-control and HRV positive sample and were detected as shown by the 375 bp amplified band (Figure 4(A), lane 1 and 2). In addition, Negative control was not detected.

For the detection of HAV in environmental sample, RT-PCR amplification was carried out with standardized positive-control, HAV positive stool sample, and D.W as negative control. All of both standardized positive-control and HAV positive sample and were detected as shown by the 413 bp amplified band (Figure 4(A), lane 1 and 2). In addition, Negative control was not detected.

## Discussion

The primary cause of acute nonbacterial gastroenteritis, NoV causes symptoms such as acute diarrhea, headache, abdominal pain, vomiting, nausea, fatigue, and low-grade fever. These symptoms usually develop 24-48 h after exposure. NoV spread by the fecal-oral route after exposure to contaminated food or water is the main source of outbreaks of acute gastroenteritis [10-12]. NoV has several characteristics that facilitate its spread [13]: (1) The infectious dose is very low (approximately 18-1,000 viral particles), which allows it to spread via several routes, including droplets, person-to-person contact, and fomites, as well as environmental contamination. The low infectious dose is also reflected in the high secondary attack rates (>30%) resulting from contact with an infected individual. (2) Prolonged periods of shedding of NoV particles increases the secondary spread (3) It is stable in a wide range of temperatures (from 0° to 60°C) and under various environmental conditions (such as those found in recreational and drinking water and a variety of food items, including raw oysters, fruits, and vegetables). (4) There are many NoV strains, which means that individuals may be infected multiple times, because of incomplete cross protection and no long-term immunity. (5) Mutations occur frequently, leading to antigenic shift, and recombination creates new strains that are capable of infecting susceptible hosts.

Because of its prevalence and highly infectious nature, the development of fast and sensitive methods to detect and differentiate among strains of NoV has been a major public health issue in South Korea, as in other countries [14,15]. A variety of enzyme immunoassay (EIA) detection kits have been produced, but they have limitations with regard to specificity and sensitivity. Some are highly specific for certain NoV strains, but they are not sensitive enough to detect a wide range of NoV strains. Furthermore, the presence of virus-specific IgM antibodies in adult sera can make it difficult to interpret the results of EIA kits [16]. Serological assays also have diagnostic limitations; after exposure, several days may elapse before IgM antibody levels are high enough to be detected. In addition, it is not possible to culture human NoV, further limiting available detection methods.

RT-PCR is a technique that is widely used in research and clinical diagnosis. However, many researchers have expressed concern about the risk of false-positive and -negative results. Viral RNA extracted from stool sample has been used as the control for RT-PCR assays for the detection of NoV in South Korea. But it has the problem such as false positive by cross-contamination. False-positive results can be avoided, to a large extent, by taking measures to avoid contamination and by employing multiple negative controls.

To overcome another problem, false-negative results, caused by polymerase-inhibiting substances, incorrect PCR mixes, or defective thermal cyclers, positive controls are required [17]. To date, several internal standards prepared and used for NoV have shown some limitation, such as the difficulty to distinguish the control by gel electrophoresis. And nested PCR is currently the most sensitive diagnostic assay for the low concentration of NoV in environmental samples, but the control for nested PCR assay to detect NoV had been not available.

To solve those problems above, we designed and constructed internal standards. As reported on the previous inspection about children's acute gastroenteritis in South Korea [18], GII-4 is the most prevalent genotype (71.9%) and GI-6 is another important genotype for is second prevalence and majority among the GI genogroup. And GI-5 was reported as one of emerging genotype on the Korean groundwater sample inspection. So, the PCR positive controls targeted these three genotypes, GII-4, GI-6, and GI-5, are meaningful and practical to detect and confirm the prevalent NoV genotypes with specificity and applicability on the environmental and clinical samples.

And the two kinds of fragments, partial VP7 of HRV and VP1/P2A region of HAV were inserted not only for the distinguished size of PCR products, but also for the detection and determination of genotype of another main causing virus of acute gastroenteritis, HRV, as well as the detection of one of main infectious disease causing virus, HAV [19,20].

The standardized positive-control plasmids described herein produced transcripts of the distinguished size when they were used as templates for RT-PCR under the same conditions as environmental and clinical samples. And they showed have the advantage to overcome false-negative results of stool sample analysis due to inhibitory substances.

Thus, they are potentially a powerful means of confirming the presence or absence of NoV infection, and may be helpful when establishing standard operating procedures to face suspected epidemics. Furthermore, these standardized positive controls can be used as controls for RT-PCR assays to detect the epidemiologically important viruses HAV and HRV, making them of greater utility to public health researchers.

## Conclusions

In the current study, four types of RNA transcripts were produced from plasmids: NoV GI-5 and GII-4 capsid regions with human rotavirus (HRV) fragment insertions, and NoV GI-6 and GII-4 capsid regions with hepatitis A virus (HAV) fragment insertions.

These size-distinguishable products were used as positive controls under the RT-PCR assay conditions used to detect NoV in stool and groundwater samples. Their reliability and reproducibility was confirmed by multiple sets of experiments.

These standardized products may contribute to the reliable and accurate diagnosis by RT-PCR of NoV outbreaks, when conducted by laboratories located in different regions.

These size-distinguishable products were applied to nationwide survey a so-called "Research on the contamination levels of norovirus in food catering facilities" funded by Korea Food and Drug Administration (KFDA) in 2010. And the result of size-distinguishable products revealed the successful construction of internal standards without the false-positive and -negative issue.

## List of Abbreviations

HRV: Human rotavirus; HAV: Hepatitis A virus; NoVs: Norovirus; ORFs: Open reading frames;

## Competing interests

The authors declare that they have no competing interests.

## Authors' contributions

SGL and SHL made substantial contributions to the study design, participated in data acquisition, analysis and interpretation and drafted and revised the manuscript critically. CIS and SWP designed and conducted the experiments. WHJ and SHO designed and conducted the experiments. SYP made substantial contributions to conception and design of the experiment and in revising the manuscript. All authors read and approved the final manuscript.
